# Decades in the Making: The Evolution of Digital Health Research Infrastructure Through Synthetic Data, Common Data Models, and Federated Learning

**DOI:** 10.2196/58637

**Published:** 2024-12-20

**Authors:** Jodie A Austin, Elton H Lobo, Mahnaz Samadbeik, Teyl Engstrom, Reji Philip, Jason D Pole, Clair M Sullivan

**Affiliations:** 1 Queensland Digital Health Centre Centre for Health Services Research The University of Queensland Brisbane Australia; 2 The Office of the Chief Clinical Information Officer eHealth Queensland Brisbane Australia; 3 Social Determinants of Health Research Center School of Allied Medical Sciences Lorestan University of Medical Sciences Khorramabad Iran; 4 Dalla Lana School of Public Health University of Toronto Toronto, ON Canada; 5 Endocrinology Department, Royal Brisbane and Women's Hospital Metro North Hospital and Health Service Queensland Health Brisbane Australia

**Keywords:** real-world data, digital health research, synthetic data, common data models, federated learning, university-industry collaboration

## Abstract

Traditionally, medical research is based on randomized controlled trials (RCTs) for interventions such as drugs and operative procedures. However, increasingly, there is a need for health research to evolve. RCTs are expensive to run, are generally formulated with a single research question in mind, and analyze a limited dataset for a restricted period. Progressively, health decision makers are focusing on real-world data (RWD) to deliver large-scale longitudinal insights that are actionable. RWD are collected as part of routine care in real time using digital health infrastructure. For example, understanding the effectiveness of an intervention could be enhanced by combining evidence from RCTs with RWD, providing insights into long-term outcomes in real-life situations. Clinicians and researchers struggle in the digital era to harness RWD for digital health research in an efficient and ethically and morally appropriate manner. This struggle encompasses challenges such as ensuring data quality, integrating diverse sources, establishing governance policies, ensuring regulatory compliance, developing analytical capabilities, and translating insights into actionable strategies. The same way that drug trials require infrastructure to support their conduct, digital health also necessitates new and disruptive research data infrastructure. Novel methods such as common data models, federated learning, and synthetic data generation are emerging to enhance the utility of research using RWD, which are often siloed across health systems. A continued focus on data privacy and ethical compliance remains. The past 25 years have seen a notable shift from an emphasis on RCTs as the only source of practice-guiding clinical evidence to the inclusion of modern-day methods harnessing RWD. This paper describes the evolution of synthetic data, common data models, and federated learning supported by strong cross-sector collaboration to support digital health research. Lessons learned are offered as a model for other jurisdictions with similar RWD infrastructure requirements.

## Background

While randomized controlled trials (RCTs) have long been accepted as the gold standard in evidence-based medicine, increasingly, there is a need to evolve this practice [1]. Well-designed RCTs are ideal for investigating the safety and efficacy of an intervention in a highly controlled setting, for example, treatment effects in drug development [2]. RCTs can fail to demonstrate the effectiveness of the intervention under complex, “real-world,” dynamic conditions [3]. This can have serious cost implications for health systems when the outcomes promised under RCT conditions fail to deliver during postmarket surveillance [4]. Increasingly, health decision makers are focusing on real-world data (RWD) to deliver large-scale longitudinal insights that are actionable. RWD are collected as part of routine care in real time using digital health infrastructure [3,5]. Modern-day health research can capitalize on the benefits of RWD with a focus on translating the findings into clinical practice. Together, the findings generated through RCTs and RWD can bridge evidence gaps to support regulatory decision-making [6]. RWD “can provide valuable complementary evidence by answering important questions on treatment effects in clinical practice that are not answered by RCTs” [7]. Perspectives in medical research regarding RCTs as the only source of practice-guiding clinical evidence need to evolve. Certainly, the use of RWD for regulatory decision-making must address key considerations to ensure that the evidence generated is fit for purpose. This includes evaluation of data relevancy and quality, including accuracy, completeness, provenance, and transparency of RWD processing [8]. Steps to address these considerations are evident in the frameworks and policies emerging over the past decade, for example, to support the Food and Drug Administration (FDA) with harnessing RWD for postmarket safety surveillance [9]. Both data obtained through RCTs and RWD have their strengths and weaknesses (Textbox 1), further emphasizing a complementary approach to both methods in modern-day health research.

**Comparing data capture methods for randomized controlled trials (RCTs) versus real-world data (RWD).**

**Data capture for RCTs**

- Demonstrate efficacy under controlled conditions (internal validity)
- Describe effect and causal relationships between an intervention and an outcome
- Data collected in a controlled and scheduled manner in accordance with the clinical trial
- Collected specifically to answer a small number of questions
- Other data regarding comorbidities may be incomplete or contain recall bias
- Intervention compared to either placebo or selected alternative
- Quality assessment tools used to review risk of bias resulting from imperfect RCT methodology
- Data elements centered on a specific research question with limited longitudinal insights

**RWD**

- Demonstrate effectiveness under real-world conditions (external validity)
- Describe the association or correlation between an intervention and an outcome
- Can be used to derive causal relationships but entail strong assumptions and rigorous methods, including evaluation of the RWD relevancy and quality
- Data often offer the advantage of being available in real time or near real time (recency of data capture)
- Provide a comprehensive picture of the patient (including details of the illness and social determinants)
- The same data used for clinical care are used for research purposes, noting that RWD can be subject to other forms of bias; for example, the care received may be a function of socioeconomic resources
- No control arm or intervention compared to standard treatment or care
- Evaluation of data quality is necessary to ensure accuracy, completeness, provenance, and transparency of processing
- Data assets may offer fragmented real-world trajectories across health systems

The interest in RWD for medical research has coincided with the rapid expansion of health IT (HIT), generating vast volumes of digital data through a myriad of sources. These include electronic medical records (EMRs), personal health records, wearable devices, mobile health, registries, and administrative data (such as claims and billing activities) [10]. However, the massive amounts of data now generated across various health care systems and platforms pose challenges in data integration and interoperability. The European Commission’s funding initiatives, such as Horizon Europe and the Innovative Health Initiative, emphasize the importance of cross-sector collaboration and data integration to foster improved interoperability and advance health care research [11,12]. Other challenges faced by RWD capture for research include privacy and confidentiality concerns [13]. Using RWD for research requires the secondary use of the data for purposes other than those for which they were originally collected [14]. Ethical and governance considerations must reflect both social license and privacy-protecting regulations. However, a difficulty faced by researchers in the digital era is conforming to regulatory frameworks established before digitization. While efforts are underway to integrate access to RWD for secondary use into updated legislation, novel methods are necessary to harness “big data” for digital health research. The same way that drug trials require infrastructure such as research nurses to support their conduct, digital health and the use of RWD also have research infrastructure needs [15]. These are not yet present in most academic institutions.

Health care research urgently requires the transformative power of data and HIT. Solutions are emerging to capture RWD siloed across HIT systems while addressing critical challenges such as interoperability, privacy, security, and effectiveness. This paper describes the rapid evolution of the medical research landscape and the ongoing development of modern-day research infrastructure. Such methods include common data models (CDMs) [16], federated learning (FL) [17], and synthetic data generation [18] supported by strong cross-sector collaboration. These novel methods are explored and, in turn, lessons learned are offered as a model for other jurisdictions with similar RWD infrastructure requirements.

## Methodology

Health data collection methods have undergone significant evolution alongside the widespread adoption of HIT systems, EMRs, and other digital health technologies. To comprehensively understand this evolution, we conducted a review and perspective study, tracing the progression from traditional data capture methods such as RCTs to the integration of RWD into medical research. Our objective was to provide both a retrospective examination and a forward-looking perspective on the evolution of research infrastructure for digital health over the past 25 years. In our methodology, we outlined the trends and strategies identified through the rapid review to overcome barriers to using RWD and enhance health research infrastructure. We emphasized the incorporation of all available health data resources to ensure a comprehensive analysis, with continued attention to data privacy, ethical compliance in digital health, and mitigation of disclosure risk.

## The Right Data for the Right Problem

### Overview

**Figure 1:**
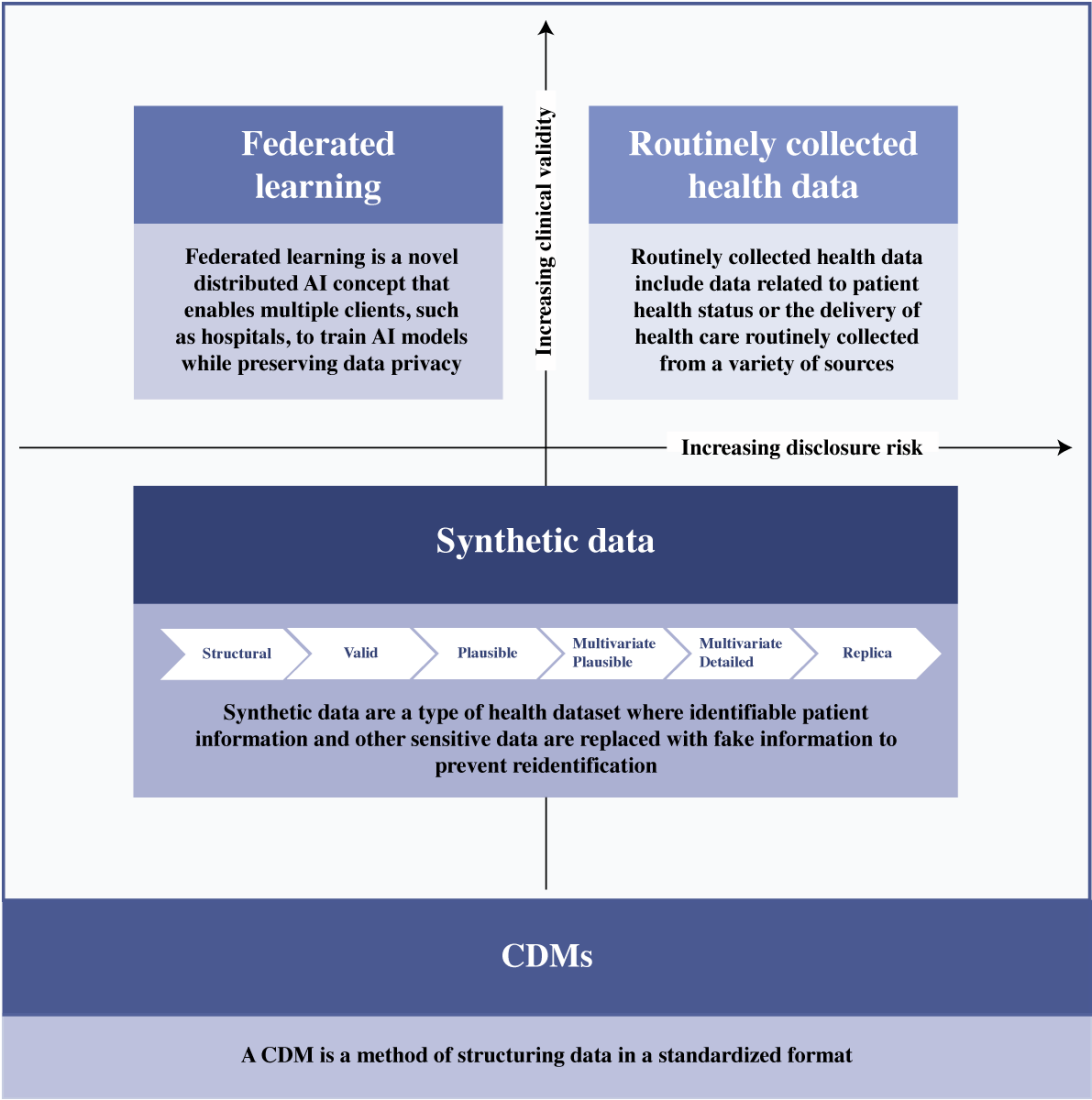
Approaches to accessing data for modern-day health research. AI: artificial intelligence; CDM: common data model.

To support the evolution of modern-day digital health research, a multifaceted approach, including synthetic data generation, mapping to CDMs, FL, and enablers to promote RWD extraction for research, is proposed. Figure 1 conceptualizes such an approach using CDM frameworks to support access to routinely collected health data, synthetic data generation, and FL infrastructure. Such an approach provides flexibility, offering the right data for the right problem at hand. Scenarios will always exist in research that require the extraction of identifiable or potentially reidentifiable patient information from data repositories for research purposes. In such circumstances, while the clinical validity of the data is high, so, too, can be the disclosure risk. Strict adherence to ethics and governance research protocols is essential. However, in recent years, there has been growing interest in alternative methods to harness RWD while minimizing disclosure risk. Methods to support RWD access in a deidentified manner, standardizing terminologies and mitigating the need for data sharing outside of enterprise structures are of particular focus. In doing so, the need to access identifiable or potentially reidentifiable patient health care data is minimized. The strategies identified to deliver each alternative method, balancing privacy concerns against clinical usefulness, are outlined in Figure 1.

### Goal 1: Synthetic Data Generation

Historically, accessing RWD has been associated with many challenges, such as laborious data access and consent procedures [19], particularly in environments in which privacy protection is prioritized and public scrutiny of digital privacy is rising [20]. Synthetic datasets, generated by a model to represent essential aspects of RWD [21], have been proposed to offer a solution for both privacy concerns and the need for widespread data access for analysis [22].

Synthetic datasets are generally classified into 3 broad categories: fully synthetic, partially synthetic, and hybrid [23]. Fully synthetic datasets entirely synthesize data without original values, ensuring privacy but compromising data validity [24-26]. In contrast, partially synthetic datasets replace selected attributes with synthetic values to preserve privacy while retaining original data, which is beneficial for imputing missing values [24-26]. Hybrid synthetic datasets combine original and synthetic data for strong privacy preservation, increasing data validity to help achieve a balance between privacy and fidelity [24-26]. However, there is a more detailed classification by the UK Office for National Statistics, which describes synthetic data in 6 levels [27], as shown in Figure 1. On the basis of this classification, a synthetic structural dataset (lowest level), developed solely from metadata, lacks clinical value and disclosure risk but is suitable only for basic code testing [27]. Conversely, a replica-level synthetically augmented dataset (highest level), which preserves format, structure, and patterns, offers high analytical value but increases disclosure risks due to its similarity to the original data [27]. The selection of synthetic data would depend on the nature of the application.

The use of synthetic data has a long-standing history dating back to the early stages of computing [28]. The early foundational work of Stanislaw Ulam and John von Neumann in the 1940s, particularly focusing on the Monte Carlo simulation technique [29], is one such example. However, the notion of fabricating synthetic data to ensure valid statistical inferences and uphold disclosure control was first suggested by Rubin (as cited in the work by Raghunathan [22]) as a discussion of the work by Jabine (as cited in the work by Raghunathan [22]). Over time, the generation of synthetic data has moved from the use of statistical methods (eg, multiple data imputation and Bayesian bootstrap) [23] to more robust algorithms [30] due to the rise of several novel tools and services [23]. An early example is the synthetic minority oversampling technique algorithm, where synthetic data points are generated by selecting a predetermined number of neighbors for each underrepresented instance, randomly choosing some minority class instances, and creating artificial observations along the line between the selected minority instance and its closest neighbors [31]. This algorithm underwent maturation over time, leading to the emergence of several variants [32-35], which predominately focused on continuous variables but failed to identify nominal features when applied to datasets with categorical features, necessitating the creation of new labels for these attributes [36].

**Figure 2:**
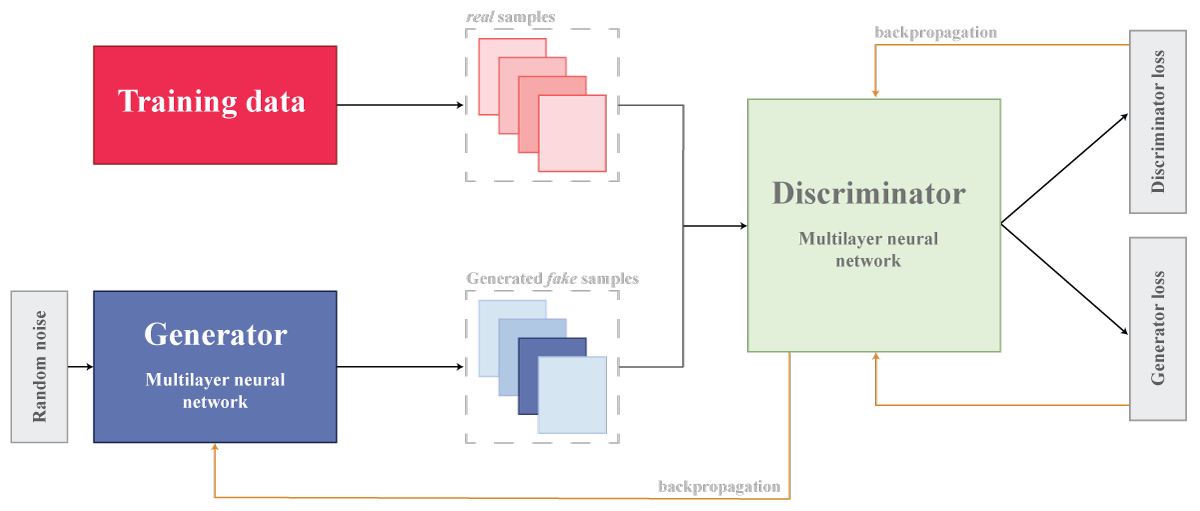
Generative adversarial network model.

The introduction of deep learning methodologies, exemplified by the inception of variational autoencoders in 2013 and generative adversarial networks (GANs) in 2014, catalyzed the evolution of more promising paradigms in the domain of synthetic data generation [37]. GANs, most importantly [37], had the potential to generate synthetic data without direct engagement with the original dataset, a feature with potential implications for reducing disclosure risk [38]. The GAN model first proposed by Goodfellow et al [38] considers simultaneously training two neural network models: (1) a generative model that captures the data distribution and (2) a discriminative model that determines where the sample is generated from the model or data distribution (Figure 2) [39]. Initially, the generative model commences with noise inputs, devoid of access to the training or original dataset, relying on feedback from the discriminative model to generate a data sample [39]. Currently, GANs have gained a lot of interest due to their capability to produce high-quality synthetic data that closely match real data, especially in health care applications [40], including (1) forecasting and planning, (2) design and evaluation of new health technology and algorithms, (3) data augmentation, (4) testing and benchmarking, and (5) education [41].

**Figure 3:**
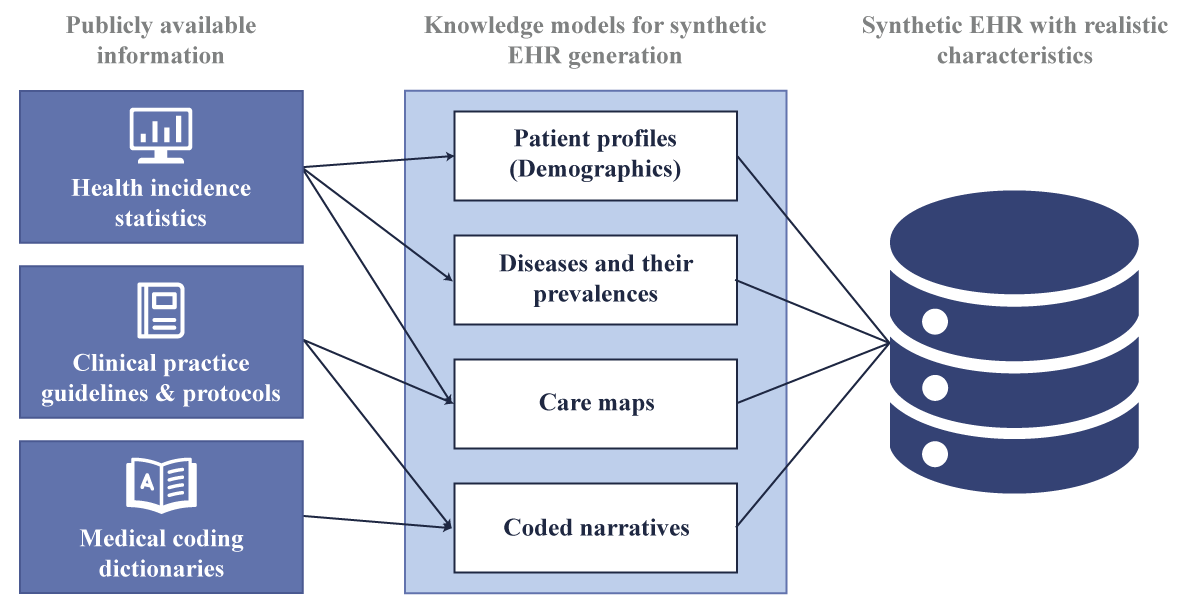
PADARSER (publicly available data approach to the realistic synthetic electronic health record) framework reproduced from Walonoski J et al [49], which is published under Creative Commons Attribution 4.0 International License [52]. EHR: electronic health record.

In the domain of published literature, GAN models are frequently discussed for their role in generating synthetic data [42-46]. However, various applications and services are now accessible for creating synthetic data tailored specifically for health care applications [23]. Among these tools are Synthea, implemented in Java; *DataSynthesizer* and *SynSys*, which are Python packages; and *synthpop* and *simPop*, both packages based on R [30,47,48]. *Synthea* uses the PADARSER (publicly available data approach to the realistic synthetic electronic health record) framework for synthetic data generation, relying on publicly available datasets instead of real electronic health records (EHRs) [49]. The framework emphasizes (1) using health statistics, (2) assuming no access to real EHRs, (3) integrating clinical guidelines, and (4) ensuring realistic properties in synthetic EHRs, as shown in Figure 3 [49].

**Figure 4:**
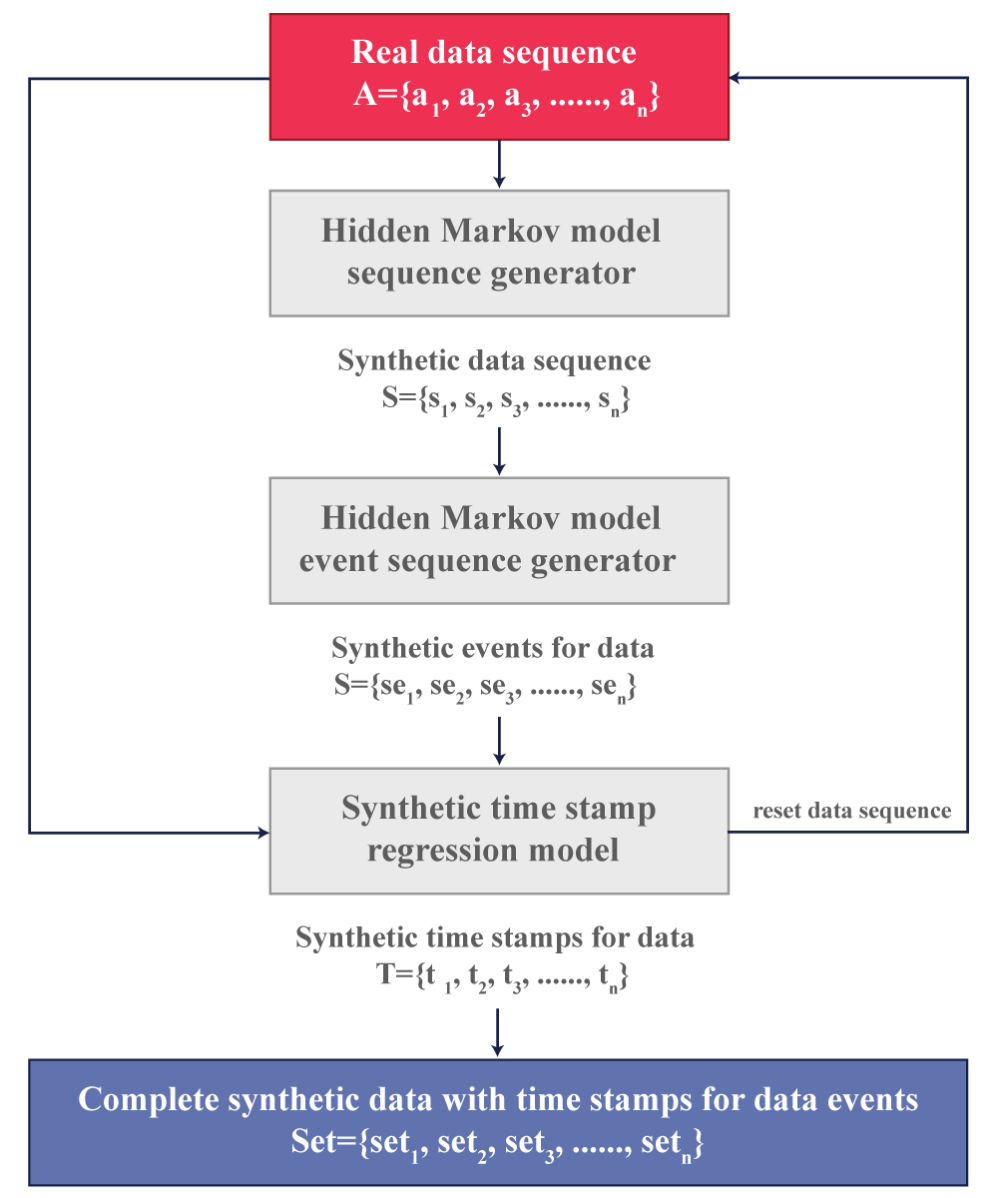
SynSys model adapted from Dahmen J et al [30], which is published under Creative Commons Attribution 4.0 International License [53].

*synthpop* uses regression trees for generating variables in a synthetic population but cannot handle complex data structures such as sophisticated sampling designs or hierarchical clusters (eg, individuals within households) [50], whereas *simPop* focuses on a modular object-oriented concept that uses various approaches, such as calibration through iterative proportional fitting and simulated annealing and modeling or data fusion through logistic regression, to generate a synthetic population [50]. In contrast, *DataSynthesizer* and *SynSys* use real patient data for the generation of synthetic datasets. For example, the *DataSynthesizer* includes 3 key modules for the generation of synthetic data: *DataDescriber*, which analyzes attribute types and distributions while preserving privacy; *DataGenerator*, which uses this analysis to create synthetic data; and Model Inspector, which provides an intuitive summary for evaluation and adjustment of parameters [51]. *SynSys* uses real data to train Markov and regression models to generate more realistic synthetic data, as shown in Figure 4 [30].

### Goal 2: CDMs

Sharing clinical data, including clinical trial data, for research is increasingly recognized as an efficient way to advance scientific knowledge [54]. However, the sharing of clinical data in health care is not without its challenges, with research highlighting concerns related to privacy, security, and interoperability [55]. While literature exists with regard to mitigating privacy and security issues in clinical data sharing for research purposes, interoperability issues persist [56]. One potential solution that has been touted to limit issues related to interoperability are CDMs [55].

CDMs are commonly used in research to enable the exchange or sharing of datasets for specific purposes [57]. The objective of a CDM is to streamline the conversion of data from diverse databases into a consistent format with standardized terminology, thereby enabling systematic analysis [58]. Over the past decade, several CDMs have been collaboratively developed and risen to the level of de facto standards for clinical research data. These include the Health Care Systems Research Network (formerly known as the HMO Research Network) Virtual Data Warehouse, the National Patient-Centered Clinical Research Network CDM, the Observational Medical Outcomes Partnership (OMOP) CDM, the Clinical Data Interchange Standards Consortium (CDISC) Study Data Tabulation Model, and the Sentinel CDM [59].

The CDISC was one of the oldest known CDMs, established in 1998, and has been pivotal in streamlining clinical data acquisition, interchange, and submission processes. With its 12 domains (Textbox 2) and unique variable naming conventions, the CDISC ensures clarity and consistency in data representation. However, it mainly aims to provide guidelines rather than imposing strict data collection requirements, allowing for flexibility for different study designs and objectives [60].

**Clinical Data Interchange Standards Consortium domains and their data structures [60].**

Demographics: 1 record per subject

Disposition: 1 record per subject

Exposure: 1 record per subject per phase or dose

Adverse events: 1 record per subject per adverse event

Concomitant medications: 1 record per subject per medication

Serum chemistry: 1 record per subject per visit per measurement

Hematology: 1 record per subject per visit per measurement

Urinalysis: 1 record per subject per visit per measurement

Electrocardiogram: 1 record per subject per visit

Vital signs: 1 record per subject per visit (per position)

Physical examination: 1 record per subject per examination, body system, or finding

Medical history: 1 record per subject per examination, body system, or condition

**Figure 5:**
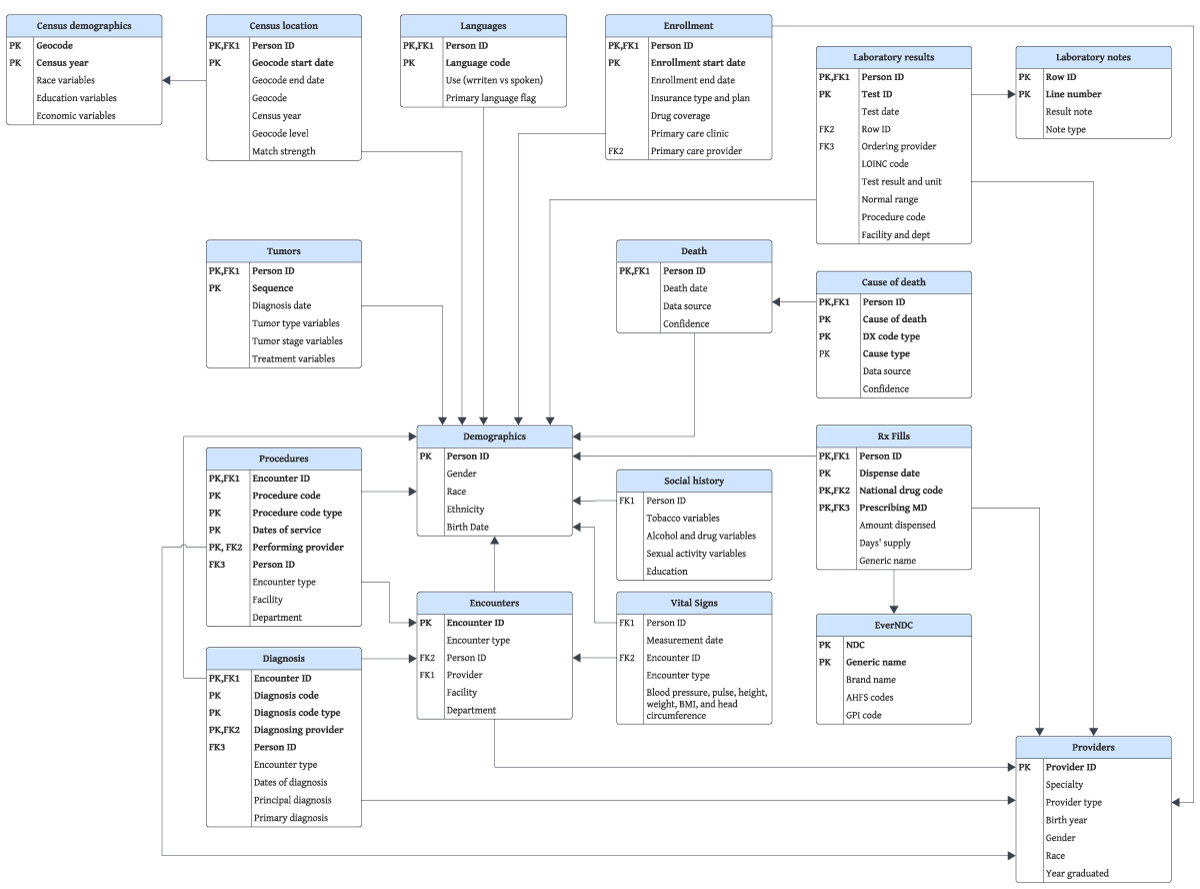
Health Care Systems Research Network Virtual Data Warehouse common data model, modified from Ross TR et al [62], which is published under a Creative Commons Attribution 4.0 International License [65]. AHFS: American hospital formulary service; DX: diagnostic; EverNDC: Ever National Drug Code; GPI: generic product identifier; LOINC: Logical Observation Identifiers Names and Codes; MD: medical doctor; NDC: National Drug Code; Rx: prescription.

Another significant CDM, Sentinel, initiated as part of the FDA’s Sentinel Initiative to monitor FDA-regulated medical products on a national scale [61]. It uses standardized concept codes with 19 tables (Textbox 3) [62], although users may need to map data due to variations in coding systems [63]. On the other hand, the Health Care Systems Research Network Virtual Data Warehouse aims to centralize data extraction and loading processes across 17 health care systems in the United States [64]. Its comprehensive structure comprises 7 content areas and >450 variables spread across 18 tables, as illustrated in Figure 5 [64], enhancing research efficiency by consolidating data management efforts [64].

**Sentinel Common Data Model [62].**

**Administrative data**

- Enrollment
- Demographic
- Dispensing
- Encounter
- Diagnosis
- Procedure
- Prescribing

**Mother-infant linkage data**

- Mother-infant linkage

**Auxiliary data**

- Facility
- Provider

**Feature engineering data**

- Feature engineering

**Registry data**

- Death
- Cause of death
- State vaccine

**Inpatient data**

- Inpatient pharmacy
- Inpatient transfusion

**Clinical data**

- Laboratory test results
- Vital signs

**Patient-reported measure (PRM) data**

- PRM survey
- PRM survey response

**Figure 6:**
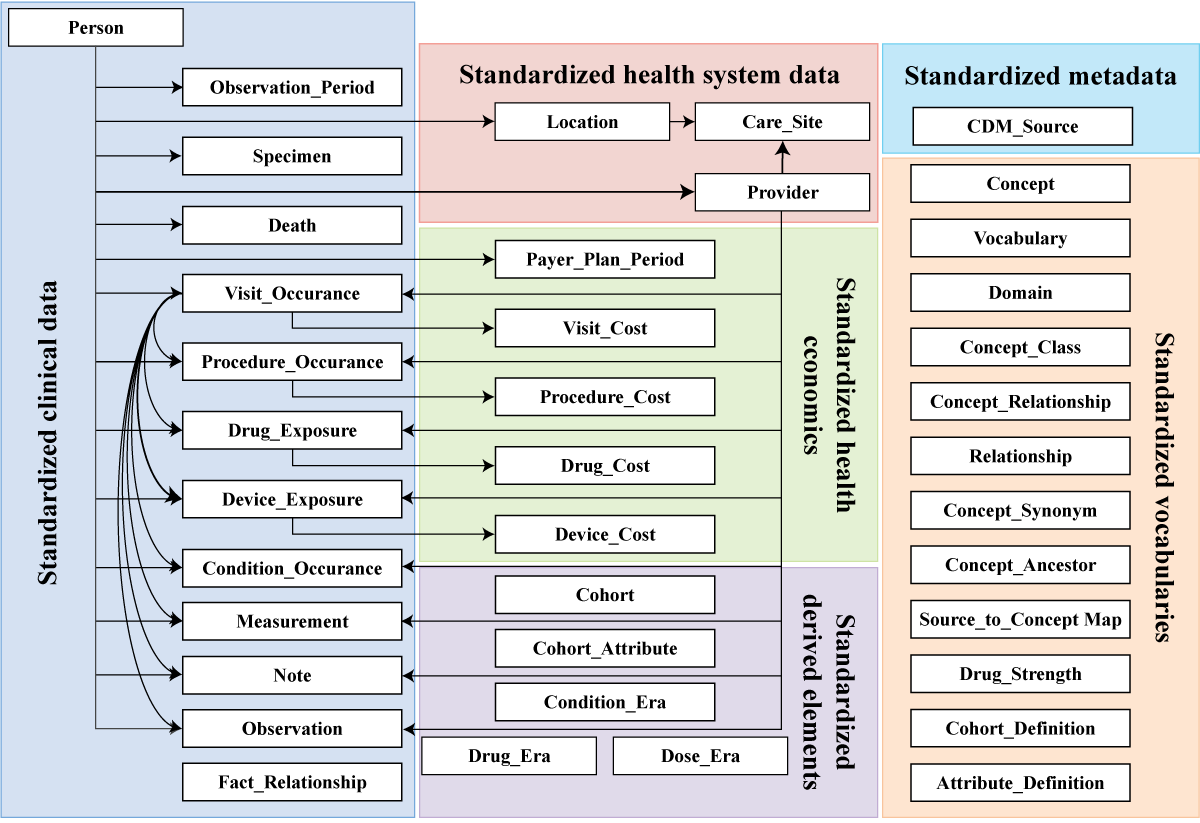
Observational Medical Outcomes Partnership Common Data Model, reproduced from Jiang G et al [72], which is published under Creative Commons Attribution 4.0 International License [52].

The National Patient-Centered Clinical Research Network was implemented to support patient-centered studies and stands out for its expansive data coverage, storing information from >100 million individuals [66] in a common format across its 23 interconnected tables [67]. It incorporates actual dates and a unique patient identifier for efficient data navigation, ensuring data integrity and facilitating comprehensive analysis [68]. In addition, the Observational Health Data Sciences and Informatics program focused on standardizing medical data representation across diverse source systems [69]. With its OMOP CDM comprising 18 tables [70], the Observational Health Data Sciences and Informatics program integrates data from >100 databases worldwide [71], addressing the need for standardized EHR data and consistent patient-level information in observational databases [69], as shown in Figure 6 [72].

**Figure 7:**
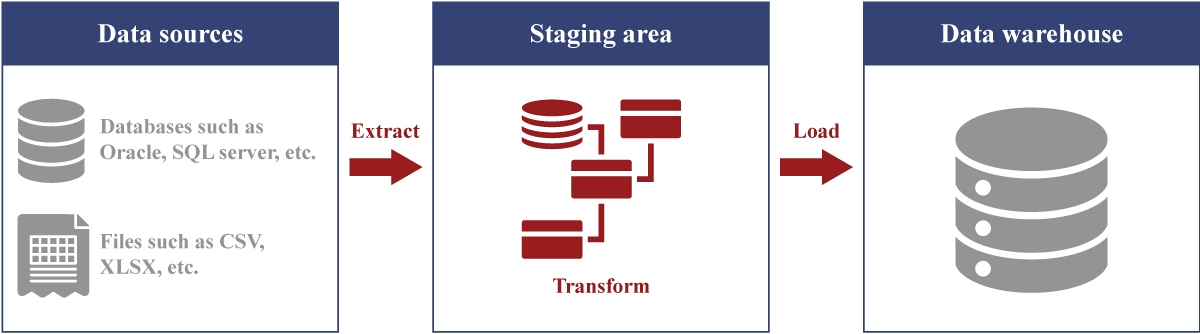
Extract, transform, and load process adapted from the work published by Abd Al-Rahman SQ et al [73], under the CC-BY-SA license [74].

While most approaches follow a different structure for storing their data, most of these models use an extract, transform, and load (ETL) process to map the data from the source database to the target structure, as shown in Figure 7 [73]. The source database may come from hospital-wide systems (such as Epic EMRs, Oracle Health, and Meditech [75]) or departmental systems (such as MOSAIQ by Elekta [76], ARIA by Varian [76], picture archiving and communication systems [77], pathology or laboratory information systems [78], and others).

The ETL process operates via 3 principal stages: extraction, transformation, and loading [79]. Extraction refers to retrieving data from relevant sources, often in file formats such as CSV [79], relational databases such as MySQL [79], nonrelational databases such as NoSQL [80], graph databases such as Neo4j [81], or accessed through Representational State Transfer clients [79]. Transformation entails the refinement and adaptation of the data to conform to the prescribed schema, encompassing tasks such as normalization, deduplication, and quality validation procedures [79]. This stage may also involve aligning the data with standardized terminologies such as the Systemized Nomenclature of Medicine–Clinical Terms or the International Classification of Diseases to ensure semantic consistency and interoperability across systems [82] or understanding preexisting standards (such as Digital Imaging and Communications in Medicine [83], the National Council for Prescription Drug Programs SCRIPT standard [84], and so on) toward mapping relevant information. Loading involves the transfer of the refined data into operational databases, data marts, or data warehouses for subsequent use [79].

### Goal 3: FL

Traditional centralized machine learning (ML) approaches face privacy and security risks [85] and limited predictive accuracy due to single-source data constraints [86]. To limit these challenges, FL has emerged as a solution by facilitating distributed model training on local devices. Google introduced FL in 2016, which uses distributed learning platforms to leverage enhanced computational abilities of devices, connect devices executing local training models, and facilitate cooperation among devices to build consensus global models of learning [87]. FL offers a secure and efficient approach to analyzing fragmented health care data [88]. This decentralized approach reduces the risk of data exposure and vulnerability to cyberattacks [89].

Over the past years, there has been a notable trend regarding how medical data are processed and used. EMRs play an important role in health care data collection and retrieval. However, strict regulations on data sharing necessitate the anonymization of sensitive patient attributes [90]. Health care organizations face challenges in aggregating clinical records for deep learning models due to privacy, data ownership, and legal concerns. Balancing data protection with leveraging collective knowledge is challenging [88]. In health care, FL initiatives are emerging as a privacy-enhancing approach to artificial intelligence and ML. These initiatives aim to collaboratively train predictive models across various institutions without centralizing sensitive personal data [91]. Recently, FL has been applied to the health care domain and life science industry, addressing the need for high-quality models in ML applications [92,93]. The FL paradigm has gained popularity for its scalable and privacy-preserving approach to joint training across federated health data repositories [85,93,94]. FL develops ML models over distributed datasets in locations such as hospitals, laboratories, and mobile devices, ensuring data privacy [88]. FL aims to overcome barriers associated with transferring sensitive clinical data to a central repository in conventional centralized artificial intelligence and ML models [85]. This approach allows for training of ML models on distributed client nodes, preserving the privacy and integrity of patient data [85]. The core concept of FL involves sharing only the parameters of the ML model being trained rather than sharing the actual data [94-96].

The FL methodology involves a network of nodes, each sharing models instead of raw training data with the central server. FL is conducted iteratively as follows. Initially, the server distributes the current global ML model parameters to all participating edge nodes. Each node then uses its locally stored data samples to update its own model based on the received parameters. Subsequently, each node transmits its updated model parameters back to the server. The server performs a global aggregation operation, combining and weighting the model parameters received from each node to generate a new set of global model parameters. This process is iterated multiple times until convergence. Importantly, at no stage do the nodes share their training data with each other or the central server, enhancing privacy and reducing bandwidth use [87,97,98].

### Goal 4: Cross-Sector Collaboration (Enablers to Promote RWD Access for Research)

The methods outlined previously provide novel approaches to RWD (or simulated RWD) access to promote digital health research. While these methods may meet most digital health research requests, access to ethically approved identifiable RWD cannot be dismissed. However, a conundrum in the digital era, with EMRs now generating vast volumes of health care data, is the limited skilled informaticians trained in data extraction and analysis. The Joint Science Academies Statement on Global Issues specific to “Digital Health and the Learning Health System” noted the basic requirement of developing and cultivating a digital health workforce, stating that “the training challenge for leveraging digital health is vast—in health care, public health and biomedical science” [99]. Those trained in data extraction are often focused on the operational activities of the health care organization. Support is needed to streamline RWD extraction for digital health research. Assigning domain experts to handle the manual data extraction steps to support researchers with access to medical RWD is necessary [100]. Academia-industry digital health collaborations can leverage uniquely skilled resources and networks to benefit both sectors [101]. Embedding staff with affiliations to both the university and health care sectors is one potential method. To overcome barriers related to university-industry collaboration, an environment fostering the missions of both sectors is necessary [102]. Being cognizant of the notable differences between the primary cross-sector objectives is necessary, for example, feasible timelines and balancing competing demands [103]. This approach is explored further in the use case below.

## Use Case

In reviewing the evolution of digital techniques used to harness RWD, consideration must be given to the application of such methods to support modern-day research. An illustrative use case is provided in this section to offer a forward-looking perspective on where such techniques may be headed.

A center dedicated to digital health research was established in Queensland, Australia. The center spanned 6 university faculties, collaborating with external government and industry partners. To overcome the challenges of harnessing RWD for research, the center established a service offering a multifaceted approach to RWD access (Figure 1). The needs and current and future state of each research infrastructure goal have been summarized in Table 1.

**Table 1:** The needs and current and future state of the research infrastructure goals of a center dedicated to digital health research established in Queensland, Australia.

	Synthetic data	CDMs^a^	FL^b^	Routinely collected health data (EMR^c^)
Needs	Access to large-scale RWDd for research, minimizing the risk of patient disclosure Support for EMR training and education Support for clinical analytics tool development Development of AIe pipelines before RWD access	Ability to collaborate on research both nationally and internationally using disparate clinical and administrative datasets Access to large-scale RWD for research, minimizing the risk of patient disclosure Reduction of burden on informaticians to run bespoke research data extracts	Promote RWD sharing across organizations while maintaining data privacy Enable model training without centralizing sensitive data, preserving individual user privacy Keep data local and share only model updates, minimizing the risk of data breaches	Support clinicians and researchers with access to RWD Reduce burden on informaticians to run bespoke research data extracts through the establishment of a dedicated team working across sectors
Current state	Semirepresentative data displaying univariant distributions sourced from publicly available health statistics	Local statewide EMR data transformed to the OMOPf CDM within the training (nonproduction) environment [104]	Established governance, ethics, and data custodian approvals Health databases relevant to a specific chronic disease use case standardized to the OMOP CDM, synthetically generated and shared with FL clients to test FL model	Established team of clinical informaticians and data engineers holding conjoint positions across both the university and health care sectors Contractual agreements established to demarcate the roles and responsibilities of the conjoint staff members accessing dual networks
Future state	Representative synthetic data mirroring multivariant distributions from the local statewide EMR	Local statewide EMR data transformed to the OMOP CDM within the production environment	Technology established with the ability to demonstrate privacy and security using synthetic data Scalable and reliable infrastructure for FL in health care designed to handle large volumes of data from diverse sources Establishment of national infrastructure for FL in digital health to generate new models of care	Continued expansion of the service to promote digital health research, including data extraction beyond the statewide EMR

^a^CDM: common data model.

^b^FL: federated learning.

^c^EMR: electronic medical record.

^d^RWD: real-world data.

^e^AI: artificial intelligence.

^f^OMOP: Observational Medical Outcomes Partnership.

The infrastructure goals highlighted in Table 1 draw upon techniques emerging in recent decades through the maturation of digital health technologies and strong cross-sector collaborations. The use case signifies how organizations are joining forces to advance modern-day research through RWD capture. No individual goal was deemed superior, yet through commitment to drive each approach to RWD access (Figure 1), this dedicated service is a method for providing researchers with the right data for the right problem.

## Discussion

### Overview

The evolution of digital health has seen many health care organizations shifting beyond the foundational levels of implementation to established methods of harnessing RWD to promote a learning health system [105]. A learning health system needs academic inquiry brought close to the routinely generated health care data, yet data security and privacy must remain paramount. While the clinical validity of the data is always greatest via direct access and extraction from the data source, so, too, is the disclosure risk. Novel methods have emerged and evolved to support access to RWD for modern-day health care research. Application of these techniques over time has provided an opportunity to reflect on the emerging needs, including the strengths and weaknesses of each goal and the future directions. In addition, the lessons learned for the described digital health research center case in point (Table 1) are included for each goal in the following sections.

### Synthetic Data Strengths, Weaknesses, and Lessons Learned

Synthetic data generation has made significant advancements in recent decades, from statistical methods to robust algorithms and established applications and services tailored to synthetic data generation for health care needs. The synthetic data created by the various models have the potential to reduce costs and accelerate data generation [106]. As such, synthetic data can have numerous applications in health care, such as estimating the impact of policies, augmenting ML algorithms, and improving predictive public health models [29]. Although synthetic data hold promise, significant work needs to be done to make them a clear option to replace RWD [107]. The reason for this conundrum is the lack of a clear understanding as to whether such a dataset can be used for decision-making or whether the final analysis would require original data [108]. Locally, the use of semirepresentative synthetic datasets (Table 1) has been effective in supporting researchers with projects less reliant on accurate representations within the synthetic data to enable research to progress while awaiting the necessary approvals to access production data. Example projects include the support of qualitative focus group sessions to co-design clinical analytics tools or development of the infrastructure for future FL projects. Work continues to explore whether similar results and accurate conclusions can be drawn from representative synthetic data when compared to RWD, with some demonstrating promising results [109,110].

Synthetic data are not free from bias [111], privacy [112], and data quality assessment [41] issues. Bias, inherent in human society, especially affects marginalized groups and is reflected in data access and generation [113]. This poses a risk with ML algorithm adoption, potentially perpetuating or amplifying societal biases [111]. Regarding privacy, while synthetic data have been claimed to be a potential solution for mitigating privacy concerns, Stadler et al [112] highlight that synthetic datasets often contain residual information from their training data, making them vulnerable to ML-based attacks that can reveal features preserved by the generative model. However, it is challenging to predict the type of information retained in synthetic data or the specific features targeted by adversaries, thereby complicating the assessment of the privacy benefits provided by synthetic data generation [112]. In addition, Stadler et al [112] explain that differential privacy, a technique used in synthetic data generation to inject noise into the original statistical information for enhanced privacy [114], provides limited defense against ML-based inference attacks, particularly for high-dimensional datasets [112]. The evaluation of data quality is another such issue, which remains an open challenge [115]. The problem arises from the absence of a standardized quality metric, which impedes fair and definitive comparisons between methods, consequently affecting the selection of an appropriate approach [41]. As a consequence of these issues, there is a crucial need for tailored regulations on synthetic data use in medicine and health care to ensure quality and minimize potential risks [116].

Synthetic data frequently reside in a regulatory gray zone concerning their use [117], and existing data protection laws such as the General Data Protection Regulation and Health Insurance Portability and Accountability Act (HIPAA) have constraints in adequately addressing all potential risks linked to synthetic data [29]. For instance, HIPAA’s privacy rule considers the creation of deidentified data as a health care operation, thus exempting them from the need for patient consent, a principle similarly applied in the General Data Protection Regulation [117]. However, synthetic health data, while not deidentified, closely replicate real data, raising questions about whether they should be classified as protected health information and require informed consent and research ethics review [117]. Some studies have demonstrated the use of synthetic data in research, eliminating the need for an ethics review [118]. Whether this is a scalable future direction for synthetic data use in research remains to be seen.

### CDM Strengths, Weaknesses, and Lessons Learned

The past 2 decades have seen the emergence of numerous CDMs to support collaborative health care research through data standardization. For example, the use of the OMOP CDM to conduct observational studies has grown extensively in recent years (from 14 publications in 2016 to 57 publications in 2020) [119], and its utility has been demonstrated in numerous, large-scale, multinational studies, such as estimating comparative drug safety and effectiveness [120-122]. The benefits are obvious for observational research in the digital era, when research questions can be addressed through combining databases with different underlying models, different information types, and different coding systems. What must not be overlooked is the potential for different biases to exist within different datasets and these nuances to be lost during translation to the CDM. Due to the complex transformations between sources and targets with varying schemas, databases, and technologies, the ETL implementations are considered prone to faults or issues [123].

According to Nwokeji and Matovu [124], these issues include complexity, cost, data heterogeneity, lack of automation, maintenance, standardization, and time. First, the growing complexity of data structures presents formidable obstacles to devising streamlined strategies [124]. In addition, the cost-intensive nature of ETL solution development imposes significant financial burdens [120]. Data heterogeneity, stemming from diverse sources and formats, further complicates the integration process [124]. Many existing ETL solutions continue to rely on manual procedures or necessitate human intervention, indicating an incomplete transition toward automation [124]. A lesson learned through the local mapping of the statewide EMR to the OMOP CDM within a nonproduction environment [104] (Table 1) highlighted the requirement for a joint clinical and technical venture. Establishing appropriate governance structures with input from clinical and technical staff is necessary to clearly articulate and endorse CDM implementation and ongoing maintenance decisions. Maintenance of ETL solutions is rendered demanding by the variety of data schemas and the dynamic nature of application requirements [124]. Furthermore, the absence of standardized methodologies for modeling ETL processes and executing workflows exacerbates these challenges [124]. Finally, the protracted process of designing, developing, implementing, and executing ETL solutions entails considerable time investments [124]. Despite these challenges, a multitude of commercial tools, including Microsoft SQL Server Integration Services, Oracle Warehouse Builder, IBM InfoSphere, and Informatica PowerCenter, alongside open-source alternatives such as Talend Open Studio and Pentaho Kettle, serve to facilitate and simplify these processes [79].

To address interoperability issues, the use of CDMs continues to expand within the health domain. Areas of future focus include the ongoing development of CDMs, their vocabularies, and tools to support their use. Further work is warranted to establish guidelines for CDM development [125] and achieving consensus on governance practices across institutions using RWD for secondary purposes [104].

### FL Strengths, Weaknesses, and Lessons Learned

Of the goals discussed, FL is the most recent technique emerging in the field of RWD access. This technology allows for learnings to be obtained from health data across organizations and locations without attempting traditional integration [87,97]. The adoption of FL in the health care domain addresses the challenges of data privacy, confidentiality, and security while still enabling efficient model training [126]. Existing works on FL in the health sector reveal a diverse range of applications categorized into prognosis, diagnosis, and clinical workflow. Prognosis-related applications encompass endeavors such as stroke prediction and prevention, brain data meta-analysis, and brain tumor segmentation [88,127,128]. Diagnosis-related applications include COVID-19 diagnosis, morphometry for Alzheimer disease, and heart disease predictions from EHRs [88,129,130]. In addition to prognosis and diagnosis, FL holds significant potential in optimizing clinical workflows within the health care sector. These applications encompass various aspects, such as drug sensitivity prediction, integration of medical data, and clinical decision support systems [88,131,132]. These advancements highlight FL in streamlining clinical workflow efficiencies, enhancing patient care, and fostering innovation in health care delivery [88]. The application of FL demonstrates its potential to enhance health care outcomes while preserving data privacy and security, highlighting the significance of interdisciplinary research and innovative solutions in advancing FL across scientific domains.

Despite the numerous advantages of FL, this methodology presents several challenges that must be addressed for its effective implementation in scientific settings. The challenges facing FL can be categorized into several critical domains. First, privacy and security concerns arise from compromised servers or clients, potentially jeopardizing data integrity and confidentiality, with active and passive attacks posing threats to overall data security [87,88,91,133,134]. The distributed nature of FL gives rise to potential new privacy and security issues that must be avoided, including the leakage of sensitive patient information (privacy) and poisoning of data (security) [135]. Second, communication bottlenecks exacerbate these challenges, hindering seamless data exchange between clients and servers and raising issues regarding network state and protocol efficacy [87,88,91]. Third, addressing the heterogeneity in data distribution poses significant challenges, particularly in handling nonindependent and non–identically distributed data [88,91,136]. Fourth, the rising computing costs, especially considering the varied capabilities of devices, highlight the critical need to address challenges related to asymmetric computing and mitigate concerns regarding energy consumption in scenarios involving on-device training [85,88,91]. Moreover, the reliability of central servers responsible for managing local training and updates is also uncertain, increasing the likelihood of data leakage and security breaches [87,88,137]. Finally, the development of new FL computing frameworks, which include redundant servers, hardware accelerators, and decentralized training models, necessitates a comprehensive and thorough investigation [87,138]. These multifaceted challenges highlight the urgent need for interdisciplinary research and innovative solutions to facilitate the successful implementation and advancement of FL across scientific domains.

FL offers a novel approach to collaborative training across health care data repositories, bypassing the need for data sharing and safeguarding sensitive medical information [139]. In the use case provided in this paper (Table 1), the process involved a combination of approaches. Standardizing the data from health databases such as EMRs and health registries via a CDM was necessary, including provision to the FL client to then test the FL model using a synthetically generated dataset. This innovative method has the potential to address various health care issues by using distributed datasets across health care facilities. By doing so, it creates opportunities for pioneering research and business opportunities in the future of health care. Researchers will focus on integrating FL into upcoming medical devices such as intelligent implants and wearables. This will lead to the development of new eHealth services, improving patient well-being.

Personalization is key in preventive health care and chronic disease management through tailored interventions. It is expected that FL will drive precision medicine and elevate health care standards in the coming years. FL also stands to transform health care delivery, offering improved precision, accessibility, and patient-centered care [85,88,97,139].

Looking ahead, challenges such as ensuring data quality and incorporating expert knowledge into FL models need attention. Designing effective incentive mechanisms is crucial to encourage users of mobile and wearable devices to participate in the FL process. This participation involves these devices collecting high-quality data locally, training local models, and sharing model updates with a central server.

### Cross-Sector Collaboration: Enablers to Promote RWD Access for Research

Digitization can accelerate RWD access through the novel technical methods emerging in recent decades. However, a holistic approach is necessary to support modern-day research in a system as multifaceted as that of digital health. The types of collaboration between the university and industry or health care sectors to drive digital transformation are varied [140]. Human factors are as important as the technologies themselves. Rybnicek and Königsgruber [141] identified 4 categories to drive the success of these cross-sector collaborations: institutional factors, relationship factors, output factors, and framework factors. The illustrative use case (Table 1) supports this approach. Embedding staff members across both types of organizations with access to both academic and health care networks and governed by the policies and procedures of the health care sector was key to supporting RWD access for research. Contractual agreements were critical to outline the key roles and responsibilities of conjoint staff, the governance frameworks by which they must abide, and clear reporting lines across both organizations. Colocation was deemed essential to build the relationship and trust. This takes both time and commitment from both sectors. As organizations continue to strive for advancements in HITs, it is the interpersonal relations that are fostering this growth. “As much as we talk about technology, at the end of the day collaboration is about people” [140].

### Conclusions

The past 25 years have seen a maturation in digital health at large. HITs are opening new and efficient ways to deliver patient care. This evolution of patient care delivery and its ability to digitally capture data through routine care has underpinned the progression of medical research techniques. A shift in perspective is necessary, moving away from the emphasis on RCTs as the only source of practice-guiding clinical evidence to include the use of RWD. Novel methods are necessary to harness the vast volumes of RWD now generated through these digital platforms. Techniques such as synthetic data generation, CDMs, FL, and collaborations between the health care and university sectors all support this common goal. Appropriate policies and frameworks are essential to address the challenges of using RWD for research. We demonstrated how, by mapping health care data to a CDM and generating a synthetic dataset, these approaches facilitate the establishment of FL infrastructure, highlighting the interoperability of these methodologies across various research environments. To achieve a learning health system, a new and disruptive research infrastructure must be established, maintained, and enhanced to expedite the translation of research findings into clinical practice. This infrastructure, equipped with emerging digital health techniques and supported by strong cross-sector collaborations, advances research by enabling more effective RWD capture, providing researchers with “the right data for the right problem.”

## Abbreviations

CDISC: Clinical Data Interchange Standards Consortium
CDM: common data model
EHR: electronic health record
EMR: electronic medical record
ETL: extract, transform, and load
FDA: Food and Drug Administration
FL: federated learning
GAN: generative adversarial network
HIPAA: Health Insurance Portability and Accountability Act
HIT: health IT
ML: machine learning
OMOP: Observational Medical Outcomes Partnership
PADARSER: publicly available data approach to the realistic synthetic electronic health record
RCT: randomized controlled trial
RWD: real-world data
